# Desquamative Inflammatory Vaginitis

**DOI:** 10.1155/S106474499600049X

**Published:** 1996

**Authors:** Jorma Paavonen

**Affiliations:** Department of Obstetrics and Gynecology University of Helsinki Helsinki Finland


Infectious Diseases in Obstetrics and Gynecology 4:257 (1996)
© 1997 Wiley-Liss, Inc.

### Images in Infectious Diseases in Obstetrics and Gynecology

*Section Editor: David E. Soper, M.D.*

### Desquamative Inflammatory Vaginitis

**Jorma Paavonen**
*Department of Obstetrics and Gynecology, University of Helsinki, Helsinki, Finland*

Desquamative inflammatory vaginitis (DIV) is an uncommon cause of purulent vaginitis in premenopausal women. DIV has also been called exudative vaginitis, hydrorrhea vaginalis, erosive vaginitis, or hemorrhagic vaginitis. DIV is thought to be an aerobic bacterial dominated syndrome caused by bacterial toxins although systematic etiologic studies have not been reported. Clinical, colposcopic, and cytological findings mimic those seen in women with trichomoniasis. However, DIV does not respond to treatment with nitroimidazoles. The diagnosis of DIV is based on clinical findings and findings on wet mount examination. Most common symptoms are frothy heavy discharge. Clinical examination reveals purulent vaginitis with patchy vaginal erythema (Fig. 1). Colposcopic examination shows multiple ecchymotic spots similar to those seen in trichomoniasis (colpitis macularis) (Fig. 2). Wet mount findings are diagnostic showing heavy coccoid bacterial flora, high number of polymorphonuclear leukocytes, and parabasal cells, but no clue cells (Fig. 3). Histopathologic examination of vaginal wall biopsy shows heavy inflammation of the stroma with capillary dilatation (Fig. 4). Most patients respond to treatment with topical clindamycin cream (2%). Bacterial vaginosis, atrophic vaginitis, or erosive lichen planus can cause differential diagnostic problems.

© 1997 Wiley-Liss, Inc.

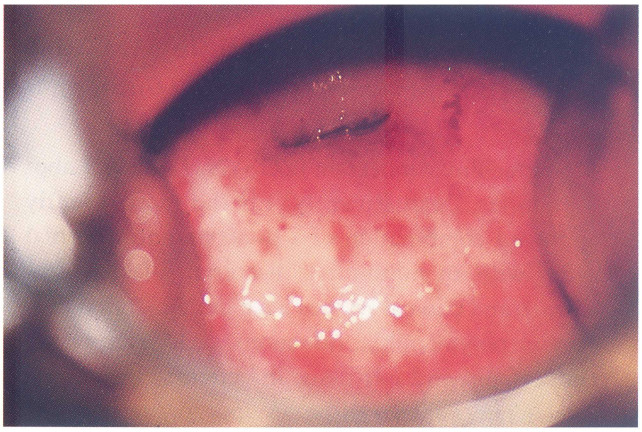

2

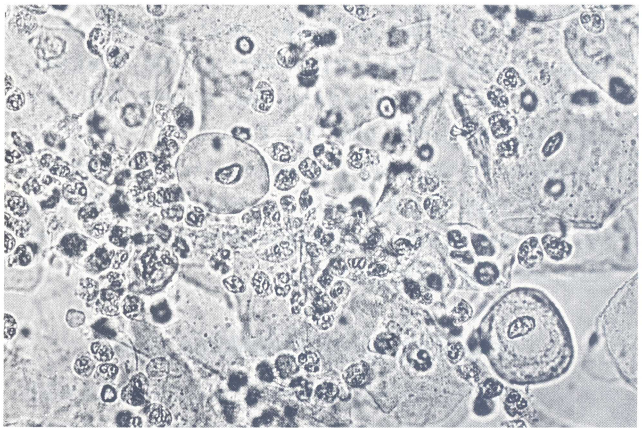

3

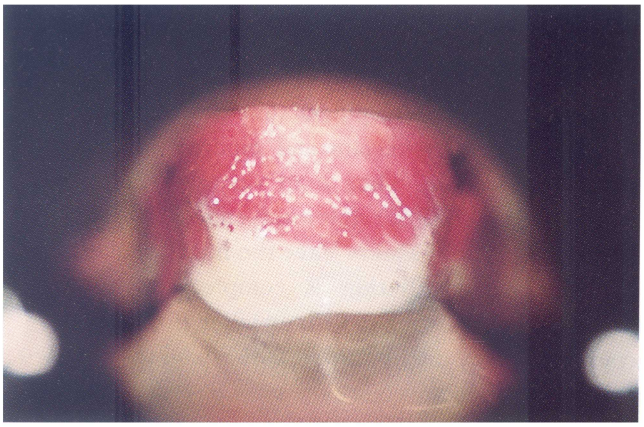

1

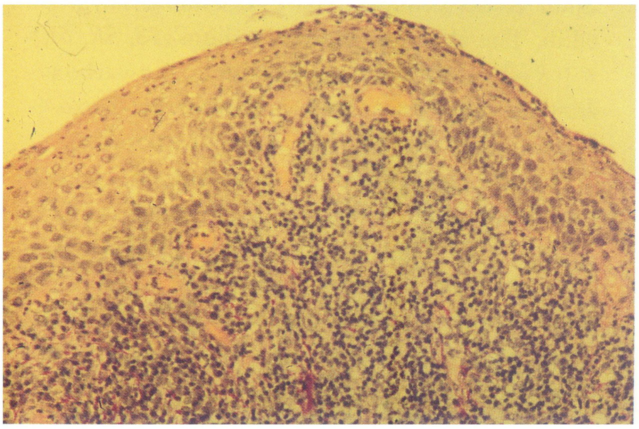

4

*Images in Infectious Diseases in Obstetrics and Gynecology* presents clinically important visual images that a practitioner in women's health might encounter. If you have a high-quality color or black-and-white photograph or slide representing such an image that you would like considered for publication, send it with a descriptive legend to David E. Soper, MD, Department of Obstetrics and Gynecology, Rush-Presbyterian-St. Luke's Medical Center, 1653 West Congress Parkway, Chicago, IL 60612-3833. *Images* is made possible through an educational grant from Pfizer, Inc.

